# Topical VEGF-C/D Inhibition Prevents Lymphatic Vessel Ingrowth into Cornea but Does Not Improve Corneal Graft Survival

**DOI:** 10.3390/jcm9051270

**Published:** 2020-04-28

**Authors:** Ann-Charlott Salabarria, Manuel Koch, Alfrun Schönberg, Elisabeth Zinser, Deniz Hos, Matthias Hamdorf, Thomas Imhof, Gabriele Braun, Claus Cursiefen, Felix Bock

**Affiliations:** 1 Department of Ophthalmology, University of Cologne, Faculty of Medicine and University Hospital Cologne, 50937 Cologne, Germany; 2 Center for Molecular Medicine Cologne (CMMC), University of Cologne, 50937 Cologne, Germany; 3 Institute for Dental Research and Oral Musculoskeletal Biology and Center for Biochemistry, University of Cologne, 50937 Cologne, Germany; 4 Department of Immune Modulation, Universitätsklinikum Erlangen, Friedrixch-Alexander-Universität Erlangen-Nuremberg, D-91052 Erlangen, Germany

**Keywords:** VEGF C/D trap, corneal transplantation, local immune modulation

## Abstract

Vascular endothelial growth factor-C/D (VEGF-C/D) regulates lymphangiogenesis. Ingrowth of lymphatic vessels is negatively associated with corneal transplantation success. In this study, we therefore analyzed the effect local blockade of VEGF-C/D has on inflamed corneas. We used the murine model of suture-induced neovascularization and subsequent high-risk corneal transplantation. Mice were treated with a VEGF-C/D trap prior to transplantation. Topical inhibition of VEGF-C/D significantly reduced lymphatic vessel ingrowth, but increased Macrophage numbers in the cornea. Furthermore, corneal transplantation success was not improved by the topical application of the compound. This study demonstrates that local VEGF-C/D inhibition is insufficient to increases corneal transplantation success, likely due to interaction with immune cells.

## 1. Introduction

Neovascularization of the cornea is associated with corneal blindness; it not only reduces vision, but also dramatically increases the rejection rate in subsequent corneal transplantations [1,2,3,4]. Corneal transplantations are the most frequently performed tissue transplantation worldwide [1,5,6]. Grafts transplanted into an avascular corneal recipient bed show a 5-year survival rate of 90%. This success rate is even achieved without HLA-matching, as commonly done with other types of transplants due to the immune privilege of the cornea [7,8]. On the other hand, grafts implanted into prevascularized recipient beds are rejected in more than 50% of cases despite immunosuppressive therapy [1,9]. Vascularization is associated with immune cell infiltration and leads to the loss of corneal immune privilege [2,3,10,11]. Several studies using the mouse model for corneal transplantation demonstrated that preventing vessel ingrowth improves subsequent corneal transplantation success [4,12,13,14,15,16,17,18]. In brief, this model involves inducing vascularization of the cornea by injury (most commonly intrastromal suture placement) accompanied or followed by interventions designed to reduce vessel content in the cornea. Approaches include the blocking of angiogenic factors [4,12,14,16,19,20,21] or mechanical disruption of vessels [17,18,22]. Corneal transplantation further increases the recruitment of immune cells such as graft infiltrating leukocytes or antigen presenting cells (APCs) including macrophages and dendritic cells (DC) into the inflamed graft [2,5]. Cells are delivered via ingrowing blood vessels and exit via lymph vessels through which they migrate towards the draining lymph nodes. Here, APCs encounter naïve T cells, which, depending on the maturation status of the APCs, become activated. Graft-infiltrating cells additionally release late chemokines and thereby guide alloreactive, activated T cells to the site of the transplant. The rejection of the graft is dependent on these T cells and the adaptive immune response often via a delayed type hypersensitivity response [3,5].

Proper characterization of lymphatic vessel involvement in the pathology of corneal transplant rejections became possible only since markers for this specific type of vessel were found. The characterization-specific surface molecules that are now used include podoplanin, Prox-1 and LYVE-1 [23].

In a case-series study, Zhen et al. found that graft rejection correlates significantly with clinically-invisible lymphatic vessel content. They concluded that lymphatic vessel content in the corneal recipient beds is a signal for a poor graft survival prognosis [9]. Similarly, Diamond et al. consistently found lymphatic vessels in rejected transplants [24]. Futhermore, it was shown that lymphatic vessels play a key role in inducing corneal graft rejection in the murine model of corneal transplantation. Graft survival was significantly improved by anti-(lymph)angiogenic therapy using anti-VEGFR3 [4].

VEGF-C and VEGF-D are known pro-lymphangiogenic growth factors. In addition, it was also discovered that the pro-angiogenic factor VEGF-A can be a pro-lymphangiogenic factor, either directly, by a proliferative effect on lymphatic endothelial cells through VEGFR-2, or indirectly, by recruitment of VEGFR1+ macrophages [25].

The VEGF-A-dependent recruitment of macrophages will further lead to the production of VEGFs (A/C/D) by these cells, which promote (lymph) angiogenesis and create a positive feedback loop that self-amplifies. Furthermore, Maruyama et al. showed in 2005 that CD11b^+^ macrophages may express LYVE-1 and Prox-1, and can form tube-like structures in the center of the cornea de novo, and thereby induce lymphangiogenesis. Depletion of these macrophages leads to inhibited lymphatic vessel growth and decreased immune cell infiltration [23]. 

The VEGF-C/D trap used in the present study is a soluble form of VEGFR-3 that potently inhibits the activity of VEGF-C & D. The molecule consists of the first three Ig-domains from the mouse VEGFR3 cloned and expressed in HEK-293 cells. This trap binds free VEGF-C and VEGF-D, and thereby prevents these angiogenic factors from signaling to the surrounding cells. It has already been shown to be a therapeutic option in the mouse model of wet AMD [26], where it inhibits choroidal neovascularization, and has also been implicated in inhibiting dry eye disease in the corresponding mouse model [27]. Here, we investigate the potential therapeutic benefit of locally-applied VEGF C/D trap in the high-risk model of corneal transplantation.

## 2. Materials and Methods

### 2.1. Animals and Anesthesia and Suture Model

All animals were treated in accordance with the institutional and national guide for the care and use of laboratory animals. 

**Figure 1:**
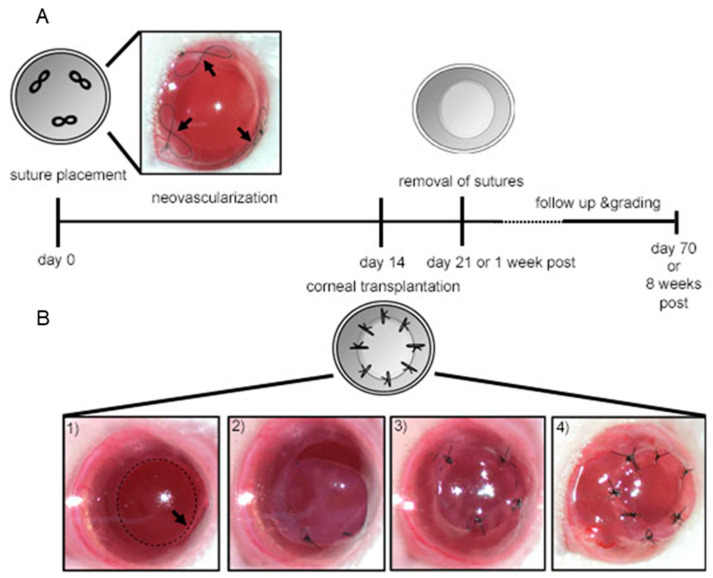
Schematic overview of suture-induced neovascularization and high-risk corneal transplantation model. (**A**) First sutures were placed into the stroma of the cornea on day 0. Sutures were left in place for 14 days. (**B**) On day 14 sutures were removed and transplantation was performed by removing central cornea of recipient and securing the donor cornea in place with eight interrupted sutures.

For suture placement and keratoplasty, we used female BALB/c mice as recipients and female C57BL/6 mice as donors as described by Yamada and Streilein [28]. Six- to eight-week-old female BALB/c mice (Charles River Germany, Sulzfeld, Germany) were anesthetized using a mixture of Ketamine and Xyalzine (120 mg/kg and 20 mg/kg body weight, respectively), and three figure-eight shaped interrupted intrastromal sutures (11-0 nylon, Serag-Wiessner, Naila, Germany) were placed in the cornea of the animals and left in place for 14 days. The sutures were placed near the limbus and spaced at equal distance to provoke a standardized response (Figure 1A). This follows the procedure of the well-established animal model to analyze corneal neovascularization [14]. VEGF-C/D trap was applied as eye drops twice daily (morning and late afternoon) for two weeks at a concentration of 200 μg/drop. The VEGF-C/D trap [29] as well as the human Fc part from human IgG1 (control), was cloned and recombinantly expressed in HEK-293 cells [30]. The first three Ig-domains from the mouse VEGFR3 (NP_032055: AA 25 – 334) protein were cloned and fused to a C-terminal IgG1 Fc domain. The construct was inserted into a sleeping beauty inducible expression vector.

For the model of Keratoplasty, on day 0, sutures were removed and these animals served as recipients for subsequent corneal transplantation.

Each treatment group included at least 10 animals. Exact group sizes are indicated in the figure legends. A nontreatment group was not included, as several studies have already published such data, showing the group to be redundant [4,31].

### 2.2. Corneal Transplantations

Allogeneic corneal transplantations were performed in the mouse model of high-risk keratoplasty. The procedure has been previously described [14]. In brief, donor corneas were excised and placed into phosphate-buffered saline until grafting (approximately 30–45 min). 

Recipient mice were anesthetized and 1.5 mm of the central cornea was excised. The graft was placed in the bed, secured with eight interrupted sutures (11-0 nylon, Serag-Wiessner, Naila, Germany) and left in place for 7 days (Figure 1B). Antibiotic treatment FLOXAL (Bausch&Lomb, Berlin, Germany) was administered onto the graft surface and the eyelids were sutured shut using 8-0 nylon (Serag-Wiessner, Naila, Germany) for 3 days.

Grafts were examined and graded for opacity twice a week until the end of the experiment. Grafts were scored by the same researcher every time. To avoid biases, treatment groups were blinded during scoring. Clinical scores were assigned as follows: 0, clear; 1, minimal, superficial (nonstromal) opacity; pupil margin and iris vessels readily visible through the graft; 2, minimal, deep (stromal) opacity; pupil margins and iris vessels visible; 3, moderate stromal opacity; only pupil margin visible; 4, intense stromal opacity; only a portion of pupil margin visible; and 5, maximum stromal opacity; anterior chamber not visible. Grafts with opacity scores of 2 or greater after 2 weeks were considered to have been rejected.

### 2.3. Flow Cytometry

Ipsilateral submandibular lymph nodes were considered draining lymph nodes of the eye and were harvested eight weeks post corneal transplantation. Organs were mechanically disrupted, pressed through 40 µM strainer and subjected to Red blood cell lysis buffer. Cell suspensions were washed with Flow cytometry Buffer containing 1% FBS and 20 µM HEPES.

Cell were stained extracellularly for CD3 (Pe-Cy7, Biolegend, Koblenz, Germany), CD4 (APC, eBioscience, Darmstadt, Germany), CD8 (APC-Cy7, Biolegend, Koblenz, Germany), CD11c (PE, Biolegend, Koblenz, Germany), CD11b (Pe-Cy7 Biolegend, Koblenz, Germany) and CD45 (APC, Biolegend, Koblenz, Germany) for 30 min on ice. Samples were then fixed and permabilized using eBioscience Fix/Perm kit and stained for Foxp3 (FITC, eBioscience, Darmstadt, Germany). Data were acquired by Millipore (Guava Incyte, Darmstadt, Germany) or Canto I (BD Bioscience, Heidelberg, Germany) and analyzed using FlowJo (v.10, Ashland, Oregon, USA).

### 2.4. Immunohistochemistry of Neovascularization and Immune Cell Infiltrate

For analyses of corneas and grafts, whole-mounts were harvested, rinsed and fixed in acetone for 30 min. Whole-mounts were rinsed in PBS and blocked with 2% bovine serum albumin for 2 h. Staining was performed with FITC conjugated CD31 (Santa Cruz Biotechnology, Heidelberg, Germany) or unconjugated rabbit antimouse LYVE-1 (AngioBio Co., Vienna, Austria) or CD3 (ABCAM, Cambridge, UK) overnight. The next day, the corneas were washed in PBS, stained with anti-rabbit Cy3 (Jackson Immuno Research, Hamburg, Germany), washed and mounted onto slides using fluorescence mounting media (DAKO, Santa Clara, CA, USA).

Quantification analysis was performed using digital images taken with Olympus BX53 microscope (Olympus, Hamburg, Germany) and Cell^f Software (Olympus, Hamburg, Germany) as previously described. In brief, CD31 positive structures were defined as blood vessels, while Lyve-1 positive structures bigger than 300 Pixel were defined as lymphatic vessels. Vessel coverage in areas were quantified and normalized to total cornea area to obtain percentage coverage. For LYVE-1 positive macrophages, the area of LYVE-1 positive lymphatic vessels was subtracted from the total LYVE-1+ area (including lymphatic vessels and Lyve-1+ macrophages) to obtain the area of LYVE-1+ macrophages only. This area was again divided by the average size of a single cell. Average size was determined by analyzing 100 cells manually for size. To obtain the number of cells per cornea, the total area of LYVE-1+ macrophages was divided by the average size of a single cell.

### 2.5. Purification and Analysis of Peritoneal Macrophages

Purification of peritoneal macrophages (Peritonial Exudate Cells; PECs) was performed as described elsewhere [32]. For the generation of peritoneal excudate cells, we used 6–8-week-old female C57BL/6 mice, as described [33,34]. Thioglycollate-induced PECs were collected from the peritoneal cavity 3 days after 2 mL i.p. thioglycollate injection. PECs were washed, resuspended and cultured at 37 °C in RPMI 1640 medium containing 10% fetal calf serum, 10 mmol/L HEPES, 1 mmol/L nonessential amino acids, 1 mmol/L sodium pyruvate, 2 mmol/L l-glutamine, 100 U/mL penicillin and 100 mg/mL streptomycin. After adhesion, nonadherent cells were removed by washing with culture medium, and adherent cells were then used as macrophages. Cells collected by this method were F4/80+ (>90%) and CD11b+ (>99%) [23,35]. For RNA expression analyses, cells were incubated in RPMI 1640 medium containing 20 or 200 ng/mL, as well as 2 µg/mL VEGF-C/D Trap or IgG isotype control for 24 h, followed by RNA isolation and real-time PCR.

### 2.6. RNA Isolation from Corneas or PECs and RT-PCR

Total RNA was isolated from the cornea or peritoneal exudate cells (PECs) using the RNeasy Micro Kit (Qiagen, Hilden, Germany) according to the manufacturer’s protocol. Three corneas were pooled to one tube for sample preparation in sufficient amount. Traces of genomic DNA were removed by DNase digestion with the RNase-free DNase Set (Qiagen Hilden, Germany). The concentration of final RNA was determined using Nanodrop. Subsequently, RNA was reverse-transcribed into single stranded cDNA using the First Strand cDNA Synthesis Kit (Fermentas, Darmstadt, Germany) according to the manufacturer’s protocol.

Real time PCR was performed with SsoFast EvaGreen Supermix (BioRad, Feldkirchen, Germany) and a CFX96 real-time system C1000 Thermal Cycler (BioRad, Feldkirchen, Germany), as well as specific primers (see Table 1). The levels of target gene expression were normalized to the housekeeping gene HPRT using the relative quantification (ΔΔCT) in Excel.

### 2.7. Mixed Lymphocyte Reaction (MLR)

Murine bone marrow-derived dendritic cells (BMDCs) were generated from bone marrow precursor cells which were isolated from femur and tibia of 6–8-week-old BALB/c mice and cultured for 8 days in R10 medium at a starting density of 2 × 10^6^ cells per 10-cm dish. The R10 culture medium consisted of RPMI 1640 supplemented with penicillin (100 U/mL), streptomycin (100 μg/mL), L-glutamine (2 mM), 2-ME (50 μM) and 10% heat-inactivated FCS. The R10 medium was supplemented with GM-CSF supernatant from a cell line transfected with the murine GM-CSF gene. On days 3 and 6, cells were fed with fresh 10 mL R10-medium containing GM-CSF (1:10) supernatant. On day 9, BMDC cultures were matured overnight with TNF (500 U/mL). During BMDC generation, VEGFR1/R2 trap or human IgG1 FC (IgG control) at a concentration of 100 μg/ml was present. Fresh VEGFR1/R2 trap was added with each feeding step. On day 9, titrated numbers (0–10,000 cells) of the aforementioned, matured BMDCs were cocultured with splenic cells from C57BL/6 N mice in 96-well flat bottom plates (BD Falcon, Heidelberg, Germany) for 72 h. Cell cultures of this allogeneic MLR were then pulsed with 1 µCi/well (Amersham, Darmstadt, Germany) for 8–16 h. Cultures were harvested onto glassfiber filtermats using an ICH-110 harvester (Inotech Brandon, FL, USA), and filters were counted in a 1450 microplate counter (Wallac, Jügesheim, Germany). Data are presented as supplemental figure (Supplementary Figure S1).

### 2.8. Statistical Analyses

All statistical analyses were performed with GraphPad Prism software (GraphPad Software, Inc., La Jolla, CA, USA) and are reported as mean +- SD. Data were tested for Gaussian distribution using the GraphPad Prism Column statistic option (D′Agostino-Pearson omnibus test), and Students t-test was used to assess significant differences between groups, with *p* < 0.05 being considered statistically significant. Corneal graft survival was assessed using Kaplan-Meier survival curves. Group size was determined by power analysis applied within the according, approved application for animal experiments N° 84-02.04.2016.A055 (approved by Landesamt für Natur, Umwelt und Verbraucherschutz Nordrhein-Westfalen, Germany, 2016).

## 3. Results

### 3.1. VEGF C/D Trap Specifically Inhibits Lymphatic Vessels while Affecting LYVE-1 Positive Macrophage Recruitment

VEGF C/D Trap is a soluble human vascular endothelial growth factor receptor-3 (VEGFR-3) fused with a Fc portion. It functions by trapping of VEGF-C and VEGF-D, and thereby blocking their action. This VEGFR-3 construct has already been shown to affect mouse VEGF-C/D [27].

**Figure 2:**
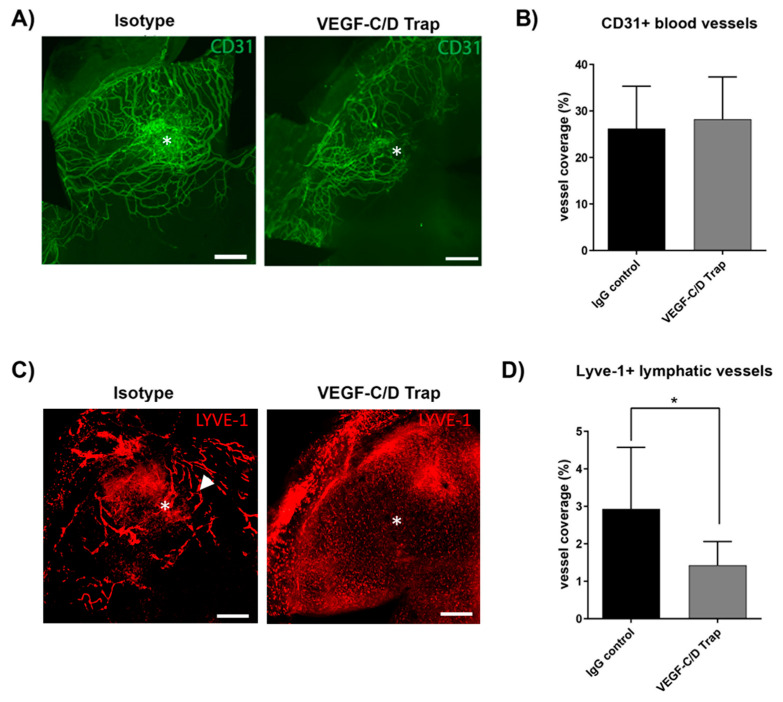
Specific inhibition of Lymphangiogenesis by VEGF-C/D blockade. (**A**) Representative examples of whole-mount corneas 14 days after suture placement stained for CD31+ vessels (green). Scale bar represents 500 μm. Asterisks indicate the position of the suture. (**B**) Quantification of whole-mount staining of CD31+ blood vessels, *p* = ns, n = 10 per group. (**C**) Representatives of same corneas as in (**A**) with staining for Lyve-1+ vessels. Scale bar represents 500 μm. Arrowhead indicate lymphatic vessels (**D**) Quantification of Lyve+1 staining, for VEGF-C/D trap (n = 10; * *p* = 0.05).

Local blockade of VEGF-C/D was achieved by local eye drop treatment 2x daily (200 µg/day) over two weeks. VEGF-C/D trap proved to be efficient in reducing lymphangiogenesis. Analyses of whole mount corneas showed no effect of the compounds on CD31+ blood vessels (Figure 2). Lyve-1+ vessels were significantly reduced in VEGF C/D Trap treated animals (*p* = 0.0145) (Figure 2D).

**Figure 3:**
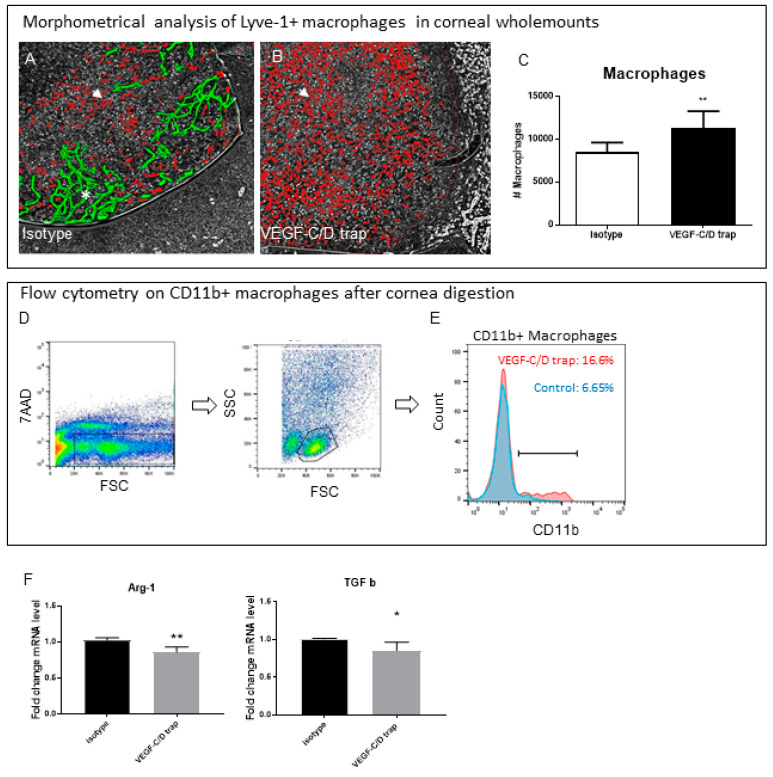
(**A**,**B**) representative images of corneal flatmounts stained with LYVE-1 and quantified by threshold analyses (red, arrowhead: macrophages, quantified; green, asterisk: lymphatic vessels, not quantified); (**C**) morphometrical analysis of Lyve-1^+^ macrophages in VEGF-C/D Trap treated vs control treated inflamed corneas (n = 10); (**D**) gating strategy: isolated cells where gated for live cells (7AAD-); lymphocyte population was identified by forward/sideward scatter plot of CD45^+^ cells; (**E**) Flow cytometry histogram of CD11b+ macrophages in VEGF-C/D trap treated corneas (red, 16.6%) in comparison to isotype control (blue, 6.6%) (representative data from 2 independent experiments; pool of 7 corneas/group); (**F**) qPCR analysis on the expression of Arginase-1 (Arg-1) and TGF-β (TGF b) in inflamed corneas treated with VEGF-C/D trap in comparison to isotype control (n = 12, in pools of 3). (* *p* = 0.05, ** *p* = 0.001).

During whole-mount analysis, we observed an increased amount of Lyve-1^+^ macrophages (Figure 3A–C). To confirm the morphological findings, we digested inflamed corneas treated with or without VEGF C/D trap. Flow cytometry also revealed an increased frequency of CD11b^+^ macrophages in the treated corneas (Figure 3D–E). To further elucidate the status of the infiltrating macrophages, we analyzed the expression of known markers for anti-inflammatory macrophages. We found a significant downregulation of the anti-inflammatory macrophage marker Arginase-1, as well as of the immune modulatory cytokine TGF-β (Figure 3F).

**Figure 4:**
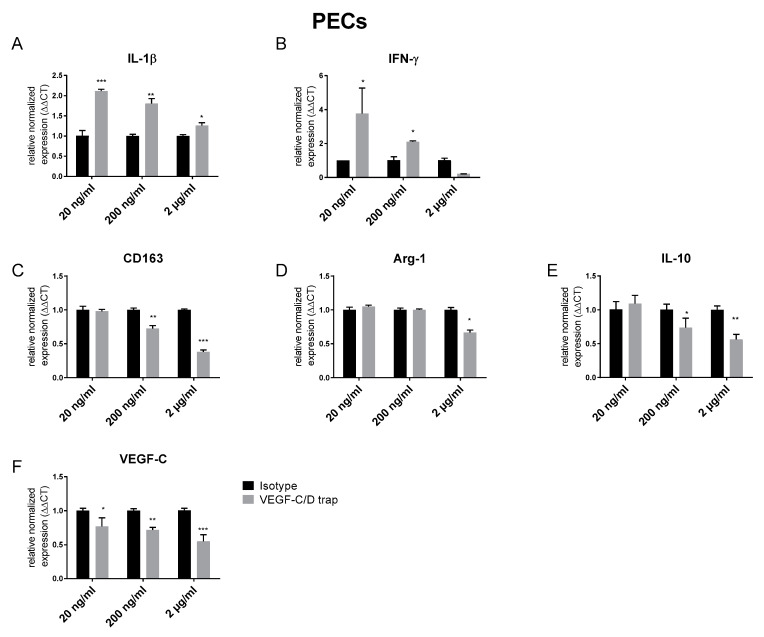
In vitor effect of VEGF-C/D Trap on pro- and anti-inflammatory markers in macrophages. (**A**,**B**) Murine Macrophages isolated from the peritoneum (Peritoneal Excudate Cells (PECs)) treated with different concentrations of VEGF-C/D Trap express increased levels of the proinflammatory cytokines IL-1β and IFN-γ; (**C**–**E**) in contrast, VEGF-C/D Trap treated PECs downregulate the immune regulatory markers CD 163 and Arg-1, as well as the immune modulatory cytokine IL-10; (**F**) VEGF-C/D Trap downregulates the expression of VEGF-C. Expression was compared with PECs treated with the same concentrations of the corresponding IgG isotype control. (n = 3, * *p* = 0.05, ** *p* = 0.001, *** *p* = 0.0001).

Based on the in vivo findings, we analyzed the direct effect of VEGF-C/D trap on macrophages in vitro. Therefore, we generated peritoneal macrophages and treated them with different concentrations of VEGF-C/D trap. By quantitative real-time PCR, we found an increased expression of the pro-inflammatory cytokines IL-1β and IFN-γ (Figure 4A,B). In line with the in vivo findings, markers of anti-inflammatory macrophages like CD163 and Arginase-1 (Arg-1), as well as IL-10, were dose-dependent downregulated (Figure 4C–E). Furthermore, we found a downregulation of the VEGF-C expression itself by VEGF-C/D trap (Figure 4F).

### 3.2. Topical VEGF-C/D Trap Has No Beneficial Effect on Survival of Cornea Transplantation

**Figure 5:**
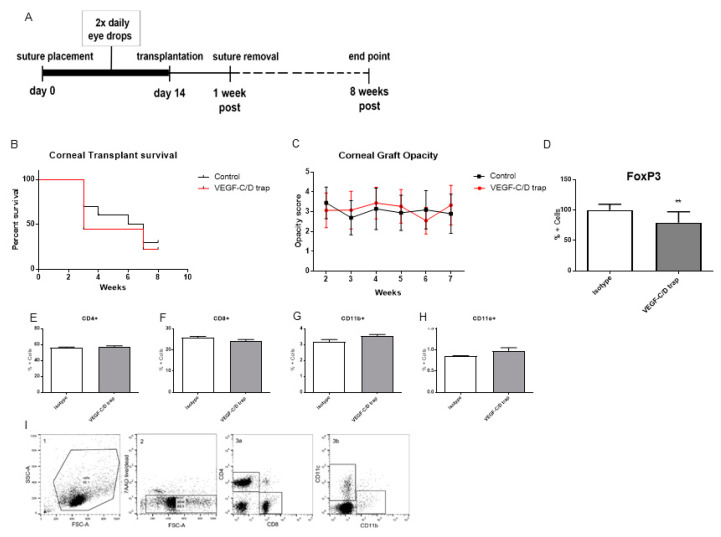
VEGF-C/D blockade did not improve corneal transplantation (**A**) Outline of Corneal transplantation model; (**B**) Corneal graft survival was not improved by VEGF-C/D trap treatment, *p* = ns, n = 10; (**C**) Opacity of corneal grafts after transplantation over time; (**D**) FoxP3+ T cell frequency was significantly reduced in the VEGF-C/D treatment group; (**E**,**F**) CD4+ and CD8+ cell numbers were not altered, *p* = ns, n = 9; (**G**,**H**) CD11b+ and CD11c+ cell frequencies were the same between groups, *p* = ns, n = 10; (**I**) Gating strategy for Immune cells: 7AAD negative cells (alive) (2), gated for for CD4 and CD8 (3a) or CD11c or CD11b (3b). (** *p* = 0.001).

In corneal transplantation, we could show that lymphatic vessels play a crucial role in determining the risk status of the recipient cornea. In addition, it was shown that the systemic application of VEGF C/D trap improves graft survival [4]. So, we evaluated the effect of topical application of VEGF-C/D trap on corneal graft survival in the corneal high-risk transplantation model. Balb/c mice were subjected to the same treatment schedule as that of the suture model. On day 14, animals were then subjected to corneal transplantations and received a 1.5 mm graft from C57B/6 mice (Figure 5A). Eight weeks post corneal transplantation we found that treatment with VEGF-C/D Trap prior to transplantation had no benefit for corneal graft survival (Figure 5B). Interestingly, we found a decreased frequency of FoxP3+ T cells in the draining lymph nodes of the transplanted eye (Figure 5D). Further immune cells (CD11b^+^, CD11c^+^, CD4^+^, and CD8^+^) in the draining lymph nodes showed no differences in frequencies between groups (Figure 5E–H).

## 4. Discussion

The involvement of lymphatic vessels in the pathogenesis of corneal transplant rejection has been demonstrated by clinical observations and several studies [1,9,10,14,24]. In the murine model of corneal transplantation and neovascularization, it was clearly demonstrated by our group that systemic treatment with an VEGFR3 blocking antibody reduced lymphatic vessel ingrowth [4]. Furthermore, we could show that the same antibody improved transplant survival to a similar degree as inhibition of both blood and lymphatic vessel did [9].

Recently, Emami-Naeini et al. used a VEGFR3-Ig fusion protein as VEGF C/D trap in the mouse model of high-risk keratoplasty. By systemic treatment, they could inhibit the growth of both blood and lymphatic vessels during inflammation and improve corneal graft survival. Interestingly, when applying the VEGF C/D trap after high-risk transplantation, they observed that only lymphangiogenesis was inhibited [36].

However, while the systemic treatment of mice can serve as proof of principle, systemic blockage of VEGF-C/D in the clinic is unfeasible, since the systemic inhibition of angiogenic factors has been shown to cause cardiovascular problems [37]. Therefore, strides have been made in recent years in the search for a more readily clinically adaptable method that locally impacts neovascularization of the cornea and can be applied topically. Local degradation or prevention of vessel ingrowth to improve corneal transplantation success has already been demonstrated in animal models. The removal of vessels by local photodynamic therapy (PDT) or fine needle diathermy, for example, has significantly improved transplant acceptance [17,18]. Both approaches, however, are invasive, can require multiple sessions and have potential long-term effects on limbal epithelial stem cell function [38]. The need for noninvasive, effective topical treatment alternatives, therefore, still exists.

Eye drops are the most desirable candidate for patient comfort and administration ease. A potential draw-back for eye drop treatment schemes is compliance, as patients are themselves responsible for administration. Nonetheless, eye drops have the significant advantage of being a non-invasive, patient-friendly administration option. Hence, in this study we opted to test locally-applied VEGF-C/D trap eye drops in this study.

Very recently, we showed that Aflibercept, a VEGFR1/R2 trap applied topical as eye drops, can inhibit corneal neovascularization, improve graft survival and induce local upregulation of FoxP3 gene expression [21]. This led us to believe that topical VEGF-C/D inhibition is a feasible approach, too. Indeed, we found that VEGF-C/D trap did exhibit a significant inhibition of lymphatic vessel growth but not blood vessel growth when delivered locally with eye drops.

However, accompanied by the reduced level of lymphatic vessels, we found an increased frequency of macrophages in the cornea. Recently, our group showed that IL-10^-/-^ mice also had a reduced lymphangiogenic response to inflammation accompanied by an increased level of CD11b+ macrophages, and developed a persistent corneal inflammation. Furthermore, treatment with IL-10 reduced the macrophage load in the inflamed cornea and induced an M2-like, anti-inflammatory status of macrophages in vitro [32]. In addition, it was shown that macrophages were responsible for the induction of corneal lymphangiogenesis [39], irrespective of the nature of the corneal damage and mouse strain [40]. Therefore, we determined whether the macrophage population in the cornea of VEGF-C/D trap treated mice were pro- or anti-inflammatory. In corneas treated with VEGF-C/D trap for two weeks during an inflammatory/proangiogenic stimulus, Arginase-1, as well as TGF-β, both indicators for anti-inflammatory macrophages, were downregulated (Figure 3F). Although the fold change of these factors was small (~1.25×), the biological relevance of these data is supported by our in vitro data. Here, PECs treated with VEGF-C/D trap showed, on the one hand, less expression of markers of alternatively activated macrophages Arginase-1, CD163 and IL10, and, on the other hand, an increased expression of the proinflammatory cytokines IL-1β and IFNγ.

It was shown that the blockade of VEGF-C/D in the skin during inflammation reduced inflammatory cell migration and inflammation resolution [26]. In line with this, we found no beneficial effect of VEGF-C/D trap when applied prior to transplantation as eye drops.

Furthermore, although not significant, we showed that antigen-presenting cells isolated from the draining lymph nodes of the VEGF-C/D trap treated mice showed an increased capacity to stimulate allogeneic T cells in a mixed lymphocyte reaction (Supplementary Figure S1). Since our results using topical VEGF-C/D blockade differed to the results presented by Emami-Naeini et al. [36], as well as to those from our own studies with an anti-VEGFR3 antibody [4,41], both of which utilized systemic administration of the therapeutic, we concluded that:

(1) Blocking VEGF-C/D during inflammation prior keratoplasty seemed to suppress not only lymphangiogenesis, but also the resolution of inflammation by the induction of anti-inflammatory macrophages and their interplay with lymphatic vessels.

(2) The time point of treatment, prior to or after corneal transplantation, is important for a successful antilymphaniogiogenic treatment. In contrast to Emami-Naeini et al., who used a low-risk transplantation setting and treated the mice after corneal transplantation, we used a high-risk transplantation setting and treated during the induction of inflammation prior to transplantation (Figure 4A). While Emami-Naeini could improve corneal graft survival by isolated inhibition of corneal lymphangiogenesis, our approach did not improve corneal graft survival, although we also achieved an isolated blockade of corneal lymphangiogenesis.

(3) Targeting VEGF-C/D or VEGFR3 to inhibit lymphangiogenesis seemed to have very different effects on other cell types like blood endothelial cells, as well as macrophages. Furthermore, the application route, local or systemically, also influenced the modulatory effect on lymphatic vessels as well as immune cells.

## 5. Conclusions

Corneal transplant rejection is mediated by a complex interplay of immunological reactions and is significantly enhanced by neovascularization in the cornea. This study showed that local application of VEGF-C/D inhibitors is an effective lymphatic vessel blocker, but cannot improve transplant survival, demonstrating that a combination of vessel blocking and immune-modulatory effects is necessary for an effective therapeutic intervention. Future studies should focus on determining the best approach of such a combination to continuous improvement of patient care.

## Figures and Tables

**Table 1:** Primer Used for Real-Time PCR.

mRNA	Sequence	Product Size (bp)	Annealing Temperature (°C)
**HPRT**	F: 5′-TTGGATACAGGCCAGACTTTGTTG-3′	163	60-63
	R: 5′-GATTCAACTTGCGCTCATCTTAGGC-3′		
**TNF-a**	F: 5′- AGGACTCAAATGGGCTTTCC-3′	63	63
	R: 5′-CAGAGGCAACCTGACCACTC-3′		
**IL-1ß**	F: 5′-GTCCTGTGTAATGAAAGACGGC-3′	176	62.4
	R: 5′-CTGCTTGTGAGGTGCTGATGTA-3′		
**IL-10**	F: 5′-CAGTACAGCCGGGAAGACAATA-3′	151	63
	R: 5′-GCATTAAGGAGTCGGTTAGCAG-3′		
**IFN-g**	F: 5′-GCTTTGCAGCTCTTCCTCAT-3′		61
	R: 5′-GTCACCATCCTTTTGCCAGT-3′		
**VEGF-C**	F: 5′-AGAACGTGTCCAAGAAATCAGC-3′	219	60
	R: 5′-ATGTGGCCTTTTCCAATACG-3′		
**Arg-1**	F: 5′-GCAGAGGTCCAGAAGAATGG-3′	126	60
	R: 5′-GTGAGCATCCACCCAAATG-3′		
**CD163**	F: 5′-GGCACTCTTGGTTTGTGGAG-3′	153	60
	R: 5′-GCCTTTGAATCCATCTCTTGG-3′		

## Supplementary Materials

The following are available online at https://www.mdpi.com/2077-0383/9/5/1270/s1, Figure S1: Increased stimulatory activity of VEGF-C/D trap treated antigen presenting cells. Stimulation response of draining lymph node immune cells (cells) to allogeneic stimulus of growth arrested C57BL/6 cells (Bl6) and addition of IL2 (n.s.; n = 3, representative data from two independent experiments).
