# Supplementary material for: Topical VEGF-C/D Inhibition Prevents Lymphatic Vessel Ingrowth into Cornea but Does Not Improve Corneal Graft Survival
Source: J Clin Med. 2020 Apr 28;9(5):1270. doi: 10.3390/jcm9051270 (PMC7287580; doi:10.3390/jcm9051270)
Supplement: Supplementary file 1 [file jcm-09-01270-s001.pdf]

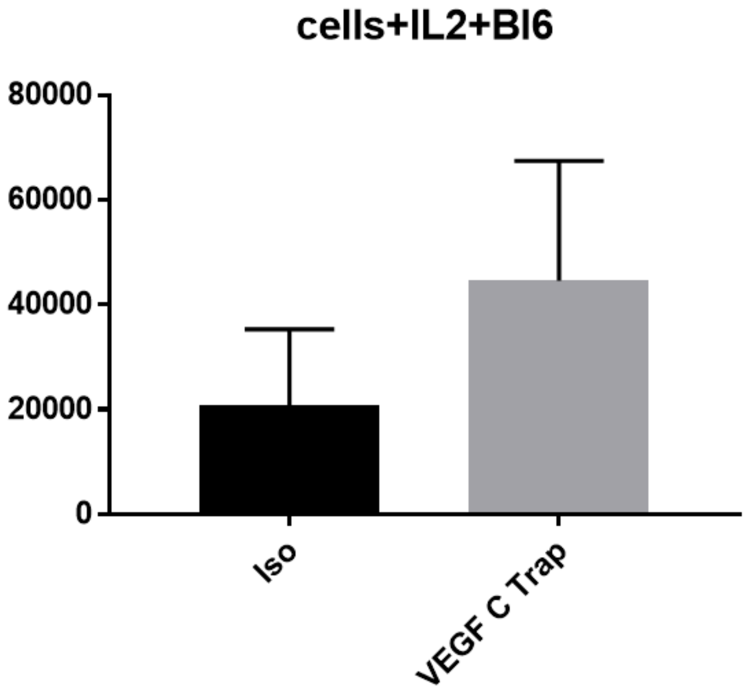

Figure S1. Increased stimulatory activity of VEGF-C/D trap treated antigen presenting cells. Stimulation response of draining lymph node immune cells (cells) to allogeneic stimulus of growth arrested C57BL/6 cells (Bl6) and addition of IL2 (n.s.; n = 3, representative data from two independent experiments).
